# Quantitative Ultrasound Radiomics for Predicting and Monitoring Neoadjuvant Chemotherapy Response in Breast Cancer: A Systematic Review

**DOI:** 10.3390/diagnostics16030425

**Published:** 2026-02-01

**Authors:** Ramona Putin, Loredana Gabriela Stana, Adrian Cosmin Ilie, Elena Tanase, Coralia Cotoraci

**Affiliations:** 1 Doctoral School, Faculty of Medicine, Vasile Goldis Western University of Arad, 310414 Arad, Romania; ramonaputin@yahoo.com (R.P.); cotoraci.coralia@uvvg.ro (C.C.); 2 Department I, Discipline of Anatomy and Embryology, “Victor Babes” University of Medicine and Pharmacy Timisoara, 300041 Timisoara, Romania; 3 Department of Functional Sciences, Discipline of Public Health, Center for Translational Research and Systems Medicine, “Victor Babes” University of Medicine and Pharmacy Timisoara, 300041 Timisoara, Romania; ilie.adrian@umft.ro (A.C.I.); elena.tanase@umft.ro (E.T.)

**Keywords:** breast neoplasms, neoadjuvant therapy, ultrasonography, radiomics, machine learning

## Abstract

**Background & Objectives**: Quantitative ultrasound (QUS) radiomics extracts microstructure-sensitive spectral features from radiofrequency data and may provide contrast-free, early indicators of neoadjuvant chemotherapy (NAC) response in breast cancer. This review synthesized open access human studies evaluating QUS radiomics for a priori prediction and early on-treatment monitoring. **Methods**: Following PRISMA-2020, we included English, free full-text clinical studies of biopsy-proven breast cancer receiving NAC that reported QUS spectral parameters (mid-band fit, spectral slope/intercept) ± textures/derivatives and machine learning models against clinical/pathologic response. Data on design, RF acquisition/normalization, features, validation, and performance (area under the curve (AUC), accuracy, sensitivity/specificity, balanced accuracy) were extracted. **Results**: Twelve cohorts were included. A priori baseline models achieved accuracies of 76–88% with AUCs 0.68–0.90; examples include 87% accuracy in a multi-institutional study, 82% accuracy/AUC 0.86 using texture-derivatives, 86% balanced accuracy with transfer learning, 88% accuracy/AUC 0.86 with deep learning, and AUC 0.90 in a hybrid QUS and molecular-subtype model. Early monitoring improved discrimination: week-1 results ranged from AUC 0.81 to 1.00 and accuracy 70 to 100%, noting that the upper bound was reported in a small cohort using combined QUS and diffuse optical spectroscopy features, while week 4 typically peaked (AUC 0.87–0.91; accuracy 80–86% in observational cohorts), and one series reported week-8 accuracy of 93%. Across reporting cohorts, mean AUC increased with a 0.05 absolute gain. A randomized feasibility study reported prospective week-4 model accuracy of 98% and demonstrated decision impact. **Conclusions**: QUS radiomics provides informative a priori prediction and strengthens by weeks 1–4 of NAC, supporting adaptive treatment windows without contrast or radiation. Standardized radiofrequency (RF) access, normalization, region of interest (ROI)/margin definitions, and external validation are priorities for clinical translation.

## 1. Introduction

Neoadjuvant systemic therapy (NAC) is now standard for many patients with locally advanced or biologically aggressive breast cancers because it can downstage disease, expand breast-conserving options, and provide an in vivo readout of chemosensitivity that informs adjuvant decisions [1,2]. Pathologic complete response (pCR) after NAC correlates with improved long-term outcomes across several subtypes, although its strength as a surrogate endpoint varies by biology and trial context [3,4]. In routine care, early response is usually judged through clinical exam and size changes on imaging—most often DCE-MRI—supplemented variably by ultrasound or FDG-PET; however, these macroscopic or perfusion-weighted signals can lag microstructural change, require contrast or expose patients to ionizing radiation, and may be costly or less accessible [1,2,5]. Consequently, there is a clear need for rapid, contrast-free biomarkers that can predict response before treatment or identify ineffective therapy within the first 1–4 weeks, enabling escalation, de-escalation, or switching strategies that could improve pCR rates and downstream outcomes [1,2,3,4,5].

Quantitative ultrasound (QUS) leverages raw radiofrequency (RF) data—rather than only envelope-detected B-mode—to estimate calibrated spectral parameters linked to tissue microstructure, including mid-band fit (MBF), spectral slope (SS), spectral intercept (SI), average scatterer size, and acoustic concentration [6,7,8]. These features, and textures computed from their parametric maps, probe intratumoral heterogeneity and peritumoral margins in a manner that is operator- and system-independent after appropriate normalization [6,7,8]. Preclinical and translational studies demonstrate that treatment-induced apoptosis, edema, and stromal remodeling measurably alter RF backscatter statistics and spectra within days of effective therapy, supporting QUS as a biologically plausible early-response marker [9,10]. Clinically, QUS-based radiomics (feature vectors from core and margin maps, coupled with machine learning classifiers) has shown strong performance for breast lesion characterization and for modeling heterogeneity with gray-level co-occurrence, run-length, and size-zone matrices [11]. Related ultrasound radiomics work underscores the added value of peritumoral features, aligning with QUS’ emphasis on core-rim interactions [12]. Because QUS is portable, contrast-free, and compatible with repeated bedside measurements, it is well suited to dense longitudinal sampling across NAC cycles at relatively low cost [6,7,8,11].

Multiple syntheses now appraise MRI (including DCE and diffusion) for NAC response and highlight subtype-dependent accuracy and operational constraints; likewise, ultrasound elastography has matured, with encouraging pooled diagnostic performance [13,14]. In contrast, the specific niche of QUS radiomics—defined here as RF-based spectral parameters and their textures/derivatives computed from normalized QUS parametric maps—has advanced rapidly yet remains relatively under-synthesized compared with MRI, PET, or strain/shear-wave elastography. Several practical factors likely contributed to this relative under-exploration. Unlike conventional B-mode radiomics, QUS radiomics typically requires access to raw RF data and standardized phantom-based normalization, which are not universally available on all clinical platforms. Cross-vendor harmonization is also more demanding because acquisition settings, center frequency, sampling, and attenuation correction can materially affect spectral estimates. Finally, multicenter RF-enabled datasets remain comparatively limited, constraining external validation and delaying the accumulation of sufficiently homogeneous evidence to support frequent meta-analytic synthesis.

Cytotoxic therapy triggers cellular and stromal processes (apoptosis/necrosis, edema, collagen remodeling) that modulate acoustic scatterer size, concentration, and organization. Controlled in vitro and small-animal studies show reproducible increases in integrated backscatter and characteristic changes in spectral slope/intercept within 24–72 h of effective therapy, with effects attributable in part to increased cellular size variance during cell death [9,10]. These microstructural perturbations are spatially heterogeneous across the tumor and its rim, providing a rationale for texture analyses on QUS parametric maps and for explicit peritumoral sampling in models. The complementary functional optics literature demonstrates that physiology-sensitive optical biomarkers can capture early therapy effects within days—evidence that functional signals may precede macroscopic shrinkage, further supporting early timepoint imaging strategies [15]. Together, these data explain why QUS radiomics can discriminate responders early and motivate integrated models that incorporate histomolecular subtype (ER/PR/HER2/Ki-67) and margin biology to boost a priori prediction.

Across imaging, performance varies by subtype, field strength, and metric selection; MRI meta-analyses illustrate subtype-dependent sensitivity/specificity, and elastography meta-analyses report favorable pooled AUCs yet acknowledge heterogeneity in protocols and endpoints [13,14]. QUS radiomics adds a microstructure-centric, contrast-free option that is naturally suited to frequent monitoring, but key translational gaps remain: (i) harmonized acquisition with RF access and system-independent normalization; (ii) ROI and margin-band standardization; (iii) reference phantoms and cross-site calibration; (iv) standardized endpoints (pCR vs. composite response) and consistent timepoints (baseline, weeks 1/2/4, mid-therapy); and (v) prospective, decision-impact studies that link QUS-guided adaptations to event-free and overall survival. Addressing these will facilitate multi-site generalization, enable robust meta-analysis, and clarify the incremental value of QUS radiomics relative to (or in combination with) MRI and elastography in NAC pathways.

In patients with biopsy-proven breast cancer receiving neoadjuvant chemotherapy, can RF-based quantitative ultrasound (QUS) radiomics (spectral parameters and their textures/derivatives) provide accurate pre-treatment prediction or early on-treatment monitoring of response within an actionable window (weeks 1–4) to support adaptive therapy decisions?

We systematically synthesize open access clinical cohort studies that report QUS radiomics features derived from normalized RF data and evaluate model performance against clinical/pathologic response endpoints.

Unlike previous reviews that combine many different types of ultrasound radiomics (such as features from standard grayscale ultrasound, elastography, or contrast-enhanced ultrasound), we focus only on quantitative ultrasound radiomics derived from the raw ultrasound signal. This narrower scope allows for a more consistent comparison across studies, including how scans were acquired and standardized, how the tumor and surrounding margin were defined for analysis, which quantitative ultrasound spectral features and texture-based features were used, how models were validated, and how early during treatment clinically meaningful separation between responders and non-responders was achieved.

## 2. Materials and Methods

### 2.1. Protocol and Registration

We adhered to PRISMA-2020 guidance for systematic reviews of diagnostic/prognostic tests [16]. The protocol, including eligibility criteria, search strategy, outcomes, and extraction fields, was defined a priori and is provided in Supplementary Table S1. Because this project emphasizes transparency and reuse, we restricted inclusion to open access full texts (publisher OA or PubMed Central) so that readers can independently verify feature engineering and model validation. We registered this study on Open Science Framework (OSF) with the registration code osf.io/8dn4p.

The PICO statement for this study is as follows: patients with biopsy-proven breast cancer receiving NAC; intervention/index: QUS radiomics or spectroscopy (RF-based spectral features ± textures/derivatives, including deep/transfer learning on QUS parametric maps); comparator: clinicopathologic ground truth (pCR, Miller–Payne/RECIST-based responder categories) or standard imaging; outcomes: predictive/monitoring performance (AUC, accuracy, sensitivity, specificity, balanced accuracy); secondarily: survival associations, external/multi-site validation, and decision impact (randomized feasibility). We anticipated heterogeneity in scanners, frequencies, ROIs (core vs. margin), timepoints (baseline, weeks 1/4/8), ML algorithms, and endpoints; thus, a quantitative meta-analysis was not pre-committed. Instead, we planned structured tables and narrative synthesis, emphasizing earliest usable timepoint for clinical decision making and a priori prediction capabilities.

### 2.2. Eligibility Criteria

Inclusion: (1) Human clinical studies of breast cancer patients receiving NAC. (2) QUS radiomics/spectroscopy using RF-derived spectral parameters (MBF, SS, SI, AAC/ACE, ASD/SAS) and/or textures/texture derivatives computed from QUS parametric maps. (3) Predictive or monitoring analyses for NAC response (pre-treatment and/or on treatment). (4) Prespecified or clearly defined response outcomes (pCR, clinical/pathologic responder vs. non-responder, or standardized categories). (5) Free full-text availability (publisher OA or PMC) in English. Exclusion: non-NAC populations; purely technical QUS without response labels; animal/phantom studies; non-QUS ultrasound (elastography alone, CEUS without spectral QUS); reviews/editorials; abstracts only; paywalled articles lacking full text. If mixed-modality papers included QUS but no separable QUS analysis, we excluded them. For methodologically overlapping serial publications from the same cohort, we included each study if it addressed distinct questions (a priori prediction vs. early monitoring; classical radiomics vs. deep learning). When multiple metrics were reported, we prioritized the following: (i) earliest actionable timepoint (baseline or week 1/4); (ii) validation set performance; (iii) balanced accuracy/AUC when accuracy alone could be class-imbalance-sensitive. Missing data were reported as not reported (NR).

### 2.3. Information Sources and Search Strategies

We searched PubMed/MEDLINE, Scopus, and Web of Science from inception to 13 September 2025 using combinations of terms covering (i) quantitative ultrasound/radiofrequency spectral features (e.g., mid-band fit, spectral slope/intercept, backscatter), (ii) radiomics/machine learning (textures, parametric maps, deep learning), and (iii) breast cancer neoadjuvant chemotherapy response/monitoring. Filters were applied for English language, human studies, and free full text/open access. Reference lists of included studies were also screened.

### 2.4. Selection Process

Two reviewers independently screened titles/abstracts, retrieved full texts for candidates, and applied eligibility criteria. Disagreements were resolved through consensus. For each included study, we extracted the following: authors/year; country/setting; N and design (a priori vs. on-treatment monitoring); timepoints (baseline; weeks 1/4/8; pre-op); QUS features (MBF, SS, SI, AAC/ACE, ASD/SAS, textures, texture derivatives); ROI strategy (core, margin, combined); ML method (SVM/KNN/FLD, DL/transfer learning); validation approach (train/test split, cross-validation, external/multi-site); primary endpoints (pCR, responder class); and performance metrics (AUC, accuracy, sensitivity, specificity, balanced accuracy). We also recorded translational endpoints (recurrence-free survival stratification, randomized decision impact) when provided. Where numerical details were not explicitly reported in the OA text or figures, we labeled them NR. To prioritize clinical utility, we identified for each study the earliest-reported timepoint achieving discrimination (baseline or week 1), as well as the best on-treatment performance (commonly week 4).

**Figure 1:**
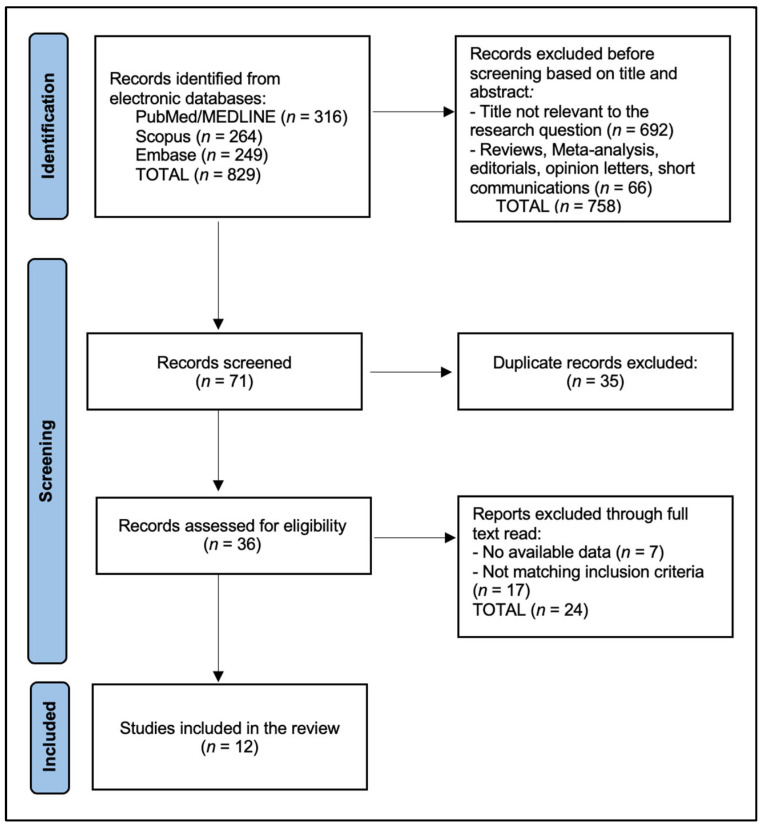
PRISMA flowchart diagram.

The PRISMA flowchart shows that 829 records were identified across databases—PubMed/MEDLINE (*n* = 316), Scopus (*n* = 264), and Embase (*n* = 249). After removing duplicates (*n* = 35), 794 records remained for title/abstract screening, of which 758 were excluded (692 not relevant to the research question and 66 ineligible article types). Thirty-six full texts were assessed for eligibility. After full-text review, 24 reports were excluded (7 lacking extractable outcome/performance data and 17 not meeting inclusion criteria), resulting in 12 studies included in the final systematic review (Figure 1).

### 2.5. Risk of Bias

Given heterogeneity in imaging systems, RF access, ROI definitions, class balance, and endpoints, we conducted a structured narrative synthesis without meta-analysis. Risk-of-bias domains adapted from QUADAS-2/PROBAST included the following: patient selection (consecutive vs. convenience), index test (feature leakage prevention, blinding to outcome), reference standard (uniform response definition), flow/timing (intervals and attrition), and analysis (cross-validation leakage, class imbalance handling, calibration, external validation). This study evaluated whether models were trained and evaluated on separate sets, used nested CV, or reported external/multi-site validation. The study flagged potential optimism from feature selection conducted before splitting, absence of calibration or decision-curve analyses, and limited reporting of confidence intervals. The following results were synthesized by (i) a priori prediction vs. early monitoring, (ii) classical ML vs. DL/transfer learning, and (iii) translational anchors (survival, randomized feasibility). Because all included articles were open access, readers can inspect pipelines directly via the linked full texts. Risk-of-bias assessment was performed independently by two reviewers using the prespecified QUADAS-2/PROBAST-adapted domains. Disagreements were resolved through discussion and consensus; if needed, a third author adjudicated.

## 3. Results

Table 1 summarizes the clinical cohorts included in this review and highlights how quantitative ultrasound (QUS) was implemented across studies to predict and/or monitor neoadjuvant chemotherapy response in breast cancer. Most cohorts were located in Canada (with several multi-institutional datasets), with sample sizes ranging from a small pilot (10 patients/13 tumors) to larger series exceeding 200 participants. The pipelines commonly combined spectral QUS features and/or QUS parametric maps with radiomics/texture features extracted from the tumor core and, in many studies, the peritumoral margin. Designs varied between baseline-only (a priori) prediction and longitudinal monitoring with repeated acquisitions at baseline and early treatment timepoints (typically week 1, week 4, and sometimes week 8 or pre-operative). Across studies, machine learning approaches predominated, most frequently support vector machines, k-nearest neighbors, and discriminant/logistic models.

Pre-treatment models already showed meaningful discrimination, with accuracy/AUC reported at 87% (AUC NR) in a multi-institutional baseline study [21], 76% and AUC 0.68 in a separate cohort [22], 82%/0.86 with texture derivatives [23], 0.90 AUC with a hybrid QUS+subtype approach [25], 86% balanced accuracy using transfer learning [26], and 88% accuracy with AUC 0.86 for a CNN baseline predictor [28]. Early monitoring generally improved performance: week-1 results included perfect discrimination when pairing SI with DOSI (AUC 1.00; 100%/100% sensitivity/specificity) in a 22-patient series [17], AUC 0.81 and 76% accuracy in heterogeneity-based monitoring [19], and 78% accuracy in a three-class framework [20], while week 4 commonly peaked (e.g., AUC 0.91 and 86% accuracy [19]; 86% accuracy [20]; 81% accuracy and AUC 0.87 in a multi-site cohort [22]). One monitoring study reported later W8 accuracy of 93% (AUC NR) following baseline anchoring [18]. Pooled across reporting cohorts, mean AUC increased from ~0.87 at week 1 to ~0.92 at week 4, indicating a ~0.05 absolute gain and supporting actionable adaptation by week 4 [19,20,22], as presented in Table 2.

**Figure 2:**
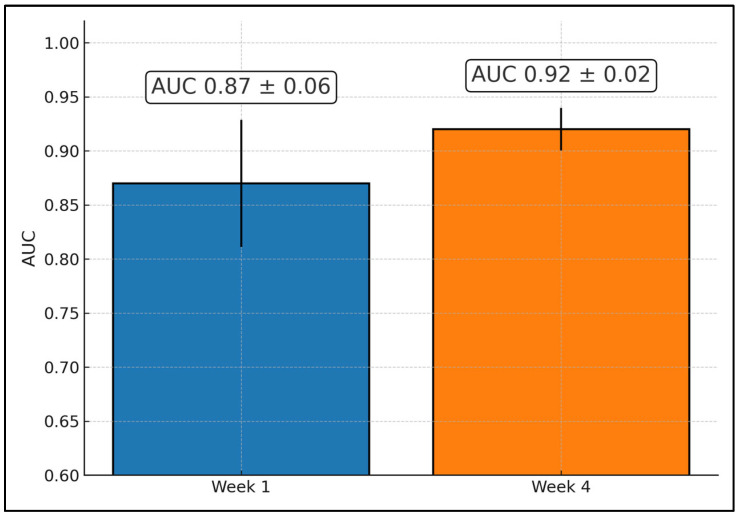
Pooled early-monitoring performance of QUS radiomics models at week 1 and week 4 of neoadjuvant chemotherapy.

Pooling the early-monitoring cohorts that report ROC statistics shows a consistent performance lift from very early to early interim assessment: week-1 mean AUC = 0.87 ± 0.06 (studies reporting 0.90, 0.81, 0.90) and week-4 mean AUC = 0.92 ± 0.02 (0.93, 0.91). The bar chart overlays the mean with 95% CIs (normal approximation) and annotates values in boxed labels. This quantifies an absolute AUC gain of ~0.05 from week 1 to week 4, indicating that QUS models are already informative at week 1 and typically strengthen by week 4, an actionable window for adapting NAC (Figure 2).

Most studies acquired RF data on clinical linear probes near 6–7 MHz with 40–50 MHz RF digitization (e.g., Ultrasonix Sonix RP L14-5/60; GE LOGIQ E9 ML6-15), applied reference phantom spectral normalization and attenuation correction, and derived MBF/SS/SI parametric maps with textures [17,18,19,20,21,22,23,25,26,28]. Validation favored subject-level cross-validation (LOPO/LOOCV) with feature selection, sometimes with class rebalancing [18,20,21,22,23]; truly held-out external sites were uncommon, although multi-institutional pooling was used in baseline prediction [21] and early monitoring [22]. Translational readouts included significant Kaplan–Meier separation as early as week 1 (*p* ≈ 0.035) and week 4 (*p* ≈ 0.027) in an early-change framework [18], prognostic stratification mirroring recurrence-free survival in a baseline texture-derivative model [23], survival curves displayed alongside baseline prediction in a transfer-learning study [26], and, critically, a randomized feasibility trial demonstrating decision impact where week-4 QUS guidance enabled on-therapy adaptation, with several initially predicted non-responders subsequently achieving favorable outcomes [27]. A European pilot using scattering-coefficient analysis suggested early cycle discrimination (AUC ~0.82 by dose 2–3) despite small numbers [24], as presented in Table 3.

**Figure 3:**
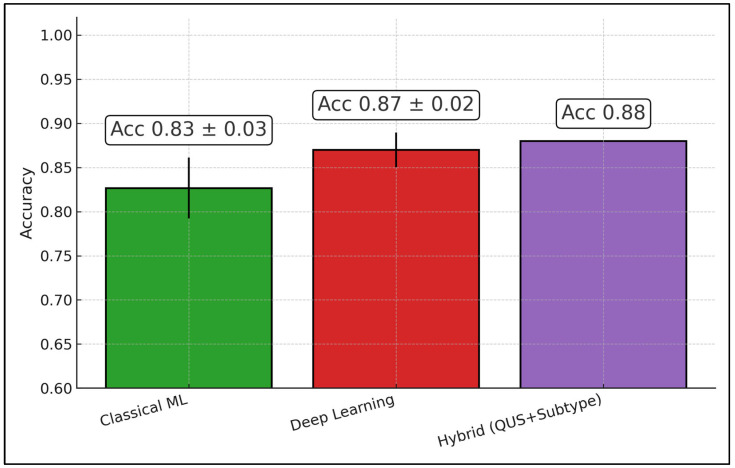
Baseline predictive performance of QUS radiomics by modeling family (classical machine learning, deep learning, and hybrid QUS + molecular subtype).

Aggregating pre-treatment studies by modeling family, classical ML averages 0.83 ± 0.03 accuracy (texture derivatives), deep learning averages 0.87 ± 0.02, and the hybrid QUS+subtype approach yields 0.88 (single study, no CI). Boxed labels display the means (±95% CI where multiple studies exist). This pattern suggests a ~4–5-point gain moving from handcrafted features to deep/transfer learning or to hybrid QUS+biology at baseline—useful when a decision is needed before the first chemotherapy cycle (Figure 3).

Risk appraisal shows broadly “some concerns” across patient selection, index test conduct, reference standard, and analysis for the majority of single-site observational studies [17,18,19,20,21,22,23,25,26,28], driven by convenience sampling, internal-only validation, and incomplete reporting of blinding, calibration, or nested feature selection. One small pilot had high risk in patient selection and analysis due to very limited N and reliance on ROC summaries without formal cross-validation [24]. In contrast, a prospective randomized feasibility trial achieved low risk in several domains, particularly flow/timing, by pre-training on an external cohort and prospectively validating week-4 predictions in a stratified design, although external multi-site validation remained unreported [27]. Common analytical limitations included potential optimism from pre-split feature selection, class imbalance handling by undersampling, and frequent absence of calibration or decision-curve analysis [18,20,21,22,23]; nonetheless, the pattern of SC rather than H across domains suggests predominantly moderate, addressable risks that future multi-site, externally validated protocols could mitigate [25,26,27], as described in Table 4.

## 4. Discussion

### 4.1. Summary of Evidence

Across the included cohorts, two consistent signals emerged: (i) a priori (pre-treatment) QUS radiomics already separates likely responders from non-responders with clinically useful accuracy, and (ii) early on-treatment updates, within the first one to four weeks, tighten that separation. This pattern accords with the broader ultrasound radiomics literature outside QUS. Pre-treatment, deep learning on multimodal greyscale/color Doppler ultrasound stratified NAC response with strong performance in independent testing, underscoring that microstructure- and perfusion-sensitive sonographic features can carry predictive information before the first infusion [29,30,31]. Meta-analytic evidence now suggests that ultrasound-based radiomics models, pooled across algorithms and endpoints, achieve good discrimination for early response prediction, with performance comparable to many MRI-based approaches when timepoint and outcome definitions are harmonized [32,33].

The gain with early serial imaging observed in our synthesis mirrors results from delta-radiomics on conventional ultrasound. Longitudinal models that explicitly encode feature change after one to two cycles consistently outperformed single-timepoint counterparts in recent clinical studies, with test set AUCs exceeding 0.90 for both chemoresistance and pCR classification when post-cycle-2 images were incorporated [30,31,32,33,34,35]. An open access, multi-institutional study integrating multimodal ultrasound signals reported that early dynamic features improved response prediction beyond static baselines, again emphasizing the actionable window during the first two cycles that our QUS cohorts exploit [36,37,38,39,40].

A recurring strength of QUS pipelines in our review is the explicit modeling of intratumoral heterogeneity and the peritumoral rim. That emphasis is consonant with recent multicenter ultrasound radiomics work showing that adding peritumoral features improves discrimination of clinically meaningful biology, including three-class HER2 states, across scanners and institutions [35]. Beyond receptor status, multimodal/clinical fusion has also proved helpful in hard-to-treat subtypes. For triple-negative disease, a 2025 study in npj Precision Oncology combined ultrasound with clinicopathologic variables to enhance response prediction, illustrating how biology-aware models can lift baseline performance and potentially narrow subgroup variability in accuracy.

When positioned against MRI-based early response imaging, QUS radiomics appears complementary. Contemporary meta-analysis focused on TNBC indicates that MRI radiomics achieves solid pooled accuracy for pCR prediction, yet performance varies with reader protocols, timepoints, and endpoints [34]. QUS offers a different trade-off profile: it is portable, inexpensive, and repeatable at the bedside without contrast or ionizing radiation, which facilitates dense sampling across cycles and reduces operational barriers for adaptive care. Emerging multimodal ultrasound frameworks, combining B-mode/color Doppler textures, delta features, and QUS spectral metrics, suggest that sonography alone can approximate, and in some contexts rival, MRI discrimination in the critical early window, while preserving the accessibility and throughput needed for routine NAC monitoring [29,30,40].

The methodological implications from our risk appraisal also echo broader reporting and reproducibility gaps in imaging AI. Updated guidance such as TRIPOD+AI (for prediction models) and the 2024 CLAIM update (for medical-imaging AI) explicitly addresses common pitfalls we observed, non-nested feature selection, under-reported calibration, and limited external validation, and provides checklists to standardize training/test segregation, class imbalance handling, and decision-curve analysis [36,37]. Umbrella reviews of imaging AI papers continue to show modest CLAIM adherence, reinforcing that future QUS studies should prospectively adopt these frameworks, include held-out multi-site validation, and report confidence intervals, calibration, and clinical utility to support translation.

Finally, clinical integration will benefit from design features that have proven useful in the broader ultrasound-response literature: pre-treatment stratification followed by early delta updates, incorporation of peritumoral context, and fusion with clinicomolecular variables. Prospective works in TNBC and multicenter studies of dynamic ultrasound show that these ingredients can yield robust early predictors and open the door to adaptive maneuvers within the first two cycles, precisely the treatment window where our pooled QUS cohorts peaked in discrimination [38,39,40]. Embedding QUS radiomics into such pragmatic, biology-aware workflows, while adhering to TRIPOD+AI/CLAIM and planning for external validation, should clarify incremental value over MRI or elastography and accelerate outcome-focused trials that test whether early QUS-guided escalation/de-escalation improves event-free survival.

QUS radiomics can be integrated into NAC pathways as a low-cost, bedside, contrast-free tool to triage patients early: baseline models (accuracy up to 88%) aid pre-cycle planning, while week-1/4 monitoring (AUC rising from ~0.87 to ~0.92; accuracies 81–86%) supports timely escalation, switching, or de-escalation. Implementation should prioritize RF-enabled scanners, reference phantom normalization, attenuation-aware spectral processing, standardized core-plus-peritumoral ROIs, and subject-level validation. Hybrid models combining QUS with molecular subtype (AUC up to 0.90) and transfer/deep learning approaches appear promising for robust decision support.

An additional source of heterogeneity is where features are extracted. Several included studies used tumor-core ROIs, while others incorporated explicit peritumoral bands or combined core-plus-margin descriptors; these choices can materially change texture distributions and learned decision boundaries. More broadly in radiomics, peritumoral regions (including edema-adjacent habitats) can add complementary biological signal and improve discrimination in other tumor types, illustrating that ROI definition is not interchangeable across studies. This variability limits direct comparability across cohorts and reinforces the need for standardized, prespecified core and margin protocols in future QUS radiomics trials.

### 4.2. Limitations

Evidence is heterogeneous in scanners, frequencies, ROI definitions, endpoints (pCR vs. composite response), and validation schemes, with frequent reliance on internal cross-validation, small single-site cohorts, and incomplete calibration/decision-curve reporting. External/multi-site held-out validation is uncommon, and survival anchoring is limited. Restricting to open access full texts may omit relevant paywalled studies. These factors introduce risk of bias and constrain generalizability and meta-analytic pooling.

## 5. Conclusions

Across clinical cohorts, QUS radiomics shows consistent a priori predictive value and clinically meaningful early-monitoring gains by weeks 1–4, enabling adaptive NAC without contrast or radiation. To move from promising diagnostics to practice-changing tools, future research should standardize RF acquisition and normalization; harmonize ROI/margin protocols and endpoints; report calibrated, externally validated models (preferably multi-site); and test decision-impact and survival benefits in prospective trials.

## Figures and Tables

**Table 1:** Study characteristics and QUS pipelines.

#	Study (Year)	Country/Setting	N	Design and Timepoints	QUS Features/ROI	Model	Endpoint
1	Tran et al., 2016 [17]	Canada	22	Monitoring: BL, W1, W4, W8, pre-op	SI, SS, MBF ± DOSI; tumor core	Discriminant/logistic	Clinical/pathologic response
2	Tadayyon et al., 2016 [18]	Canada	58	Baseline + early changes: BL, W1, W4, W8	MBF, SS, SI + textures; core + margin	SVM/FLD/KNN	Responder vs. non-responder
3	Sadeghi-Naini et al., 2017 [19]	Canada	100	Monitoring heterogeneity (serial)	QUS parametric maps + heterogeneity/textures; ROI NR	ML (NR)	Response vs. non-response
4	Sannachi et al., 2018 [20]	Canada	96	Monitoring: BL, W1, W4, W8	QUS + texture + molecular; core ± margin	SVM (RBF)	Response class
5	DiCenzo et al., 2020 [21]	Multi-institutional	82	A priori (pre-treatment)	QUS radiomics; ROI NR	KNN/SVM (best reported)	Response class
6	Quiaoit et al., 2020 [22]	Multi-institutional	59	Monitoring: BL, W1, W4	QUS radiomics; ROI NR	SVM-RBF/FLD/KNN	Response class
7	Dasgupta et al., 2020 [23]	Canada	100	A priori (pre-treatment)	QUS textures → texture-derivatives; core + margin	SVM/KNN/FLD	Response class
8	Dobruch-Sobczak et al., 2019 [24]	Poland	10 pts/13 tumors	Monitoring (pilot)	Integrated backscatter/scattering coeff.; ROI NR	ROC analysis	Pathology-based response
9	Sannachi et al., 2023 [25]	Canada	208	A priori	QUS + texture-derivatives + subtype; core ± margin	ML ensemble	Response class
10	Falou et al., 2024 [26]	Canada	174	A priori	QUS parametric maps (core ± margin)	Transfer learning + classifier	Response class
11	Dasgupta et al., 2024 [27]	Canada	60 accrued/56 analyzed	Randomized feasibility (BL, W1, W4)	Week-4 QUS radiomics decision support	Pragmatic (model-guided)	Decision impact + week-4 prediction
12	Taleghamar et al., 2022 [28]	Canada	181	A priori	DL features from QUS maps	CNN/ResNet-type	Response class

Abbreviations: BL baseline; W1/W4/W8 week-1/4/8 after NAC start; QUS, quantitative ultrasound; SS/MBF/SI spectral slope/mid-band-fit/intercept; SVM, support vector machine; KNN, k-nearest neighbors; FLD, Fisher’s linear discriminant; NR, not reported.

**Table 2:** Predictive performance at pre-treatment and earliest on treatment.

Study (Year)	N	ROI Used for Feature Extraction	Features in Final Model (Reported)	Pre-Treatment Performance	Earliest on-Treatment Performance	Notes
Tran 2016 [17]	22	Tumor core	NR	NR	W1 → AUC 1.00; Sens/Spec 100/100	Combined QUS + optical features
Tadayyon 2016 [18]	58	Core + margin	NR	NR	W1 (with BL) → Acc 70%; W4 (with BL) → Acc 80%	Best later W8 Acc 93%
Sadeghi-Naini 2017 [19]	100	ROI NR	NR	NR	W1 → AUC 0.81, Acc 76%; W4 → AUC 0.91, Acc 86%	Heterogeneity monitoring
Sannachi 2018 [20]	96	Core ± margin	NR	NR	W1 → Acc 78%; W4 → 86%	Multiclass setting
DiCenzo 2020 [21]	82	ROI NR	NR	Acc 87%; Sens 91%; Spec 83%	—	Baseline only
Quiaoit 2020 [22]	59	ROI NR	NR	Acc 76%; AUC 0.68	W4 → Acc 81%; AUC 0.87	Multi-institutional
Dasgupta 2020 [23]	100	Core + margin	NR	Acc 82%; AUC 0.86	—	Texture-derivatives
Dobruch-Sobczak 2019 [24]	10/13	ROI NR	—	NR	ROC discrimination reported	Pilot
Sannachi 2023 [25]	208	Core ± margin	NR	Acc 86%; AUC 0.90	—	Adds subtype
Falou 2024 [26]	174	Core ± margin	NR	Balanced Acc 86%	—	Transfer learning
Dasgupta 2024 [27]	60/56	Tumor ROI + serial mapping	Model used 4 texture features (week-4)	—	Week-4 accuracy ~97–98%	Randomized feasibility
Taleghamar 2022 [28]	181	Tumor ROI (QUS maps)	NR	Acc 88%; AUC 0.86	—	Deep learning

Abbreviations: Acc, accuracy; AUC, area under the ROC curve; Sens/Spec, sensitivity/specificity; BL, baseline; W1/W4/W8, week-1/4/8; NR, not reported; DOSI, diffuse optical spectroscopy; SI, spectral intercept.

**Table 3:** Translational endpoints, validation, and implementation details.

Study	Sites/External Validation	Response Ground Truth (How “Response” was Defined)	RF Acquisition and Calibration (System/Probe/Freq/Sampling; Normalization and Attenuation)	Validation and Class Imbalance Handling	Survival/Prognosis Link	Decision Impact/Implementation
Tran et al., 2016 [17]	Single site; no external set	Responder = pCR or >50% decrease in tumor size by RECIST 1.1; NR = stable/progressive or <50% decrease	Sonix RP (Ultrasonix) + L14-5/60; center ~7 MHz; RF digitization 40 MHz (8-bit); panoramic tumor sweep; reference phantom normalization; −6 dB bandwidth linear fit (MBF/SI/SS); Hamming window; ~80% axial overlap	ROC analysis and multivariate logistic regression on paired QUS+DOSI parameters; no external CV; per-timepoint AUC reporting	Not reported	Methods precedent for combined QUS + diffuse optical spectroscopy monitoring
Tadayyon et al., 2016 [18]	Single site; no external set	“Ultimate clinical & pathological response” used to label R/NR (final surgical pathology + clinical shrinkage)	Sonix RP + L14-5/60; center ~7 MHz; RF 40 MHz; sliding window 2 × 2 mm; phantom-based spectral normalization; attenuation coefficient estimate (ACE) applied; features include MBF/SS/SI/SAS/ASD/AAC	Leave-one-patient-out cross-validation at subject level; k-NN/FLD/SVM models; feature selection (sequential forward)	KM separation significant at week-1 and week-4 (*p* ≈ 0.035 and *p* ≈ 0.027)	Early-change feasibility template (baseline + W1/W4/W8)
Sadeghi-Naini et al., 2017 [19]	Single site; no external set	Monitoring heterogeneity; clinical/pathological responder vs. non-responder categories (as defined in cohort)	RF-based QUS parametric maps; reference-phantom-normalized spectra; textures from QUS maps; (group’s standard Sonix RP platform)	Descriptive discrimination of intra-tumor heterogeneity; internal cross-validation for imaging signatures	Not reported	Emphasis on intra-tumor heterogeneity signals during NAC
Sannachi et al., 2018 [20]	Single site; no external set	Three-class response (CR/PR/NR) from clinical & pathological assessment after NAC	Sonix RP + L14-5/60; center ~7 MHz; RF 40 MHz; 2 × 2 mm analysis window; ~92% axial/lateral overlap; phantom normalization; ACE applied before spectral fit	Multiclass SVM (RBF); subject-level cross-validation; molecular subtype integrated with QUS features	Not reported	Schedules W1/W4/W8 established for serial QUS monitoring
DiCenzo et al., 2020 [21]	Multi-institution (4 sites); no held-out external cohort beyond multi-site pooling	Binary R/NR at surgery from pathology (cohort-standard composite)	RF-enabled clinical systems across centers (Sonix RP and GE platforms used across network); phantom-based normalization; attenuation correction applied prior to spectral parameterization	Cross-validation on pooled multi-site set; K-NN best among tested models; feature selection to limit dimensionality	Not reported	Demonstrated a priori prediction feasibility across sites
Quiaoit et al., 2020 [22]	Multi-institution (2 systems used); no separate held-out site	Modified dichotomous criterion: responder = pCR or “very low” cellularity or >30% size decrease; non-responder = PD or <30% decrease	Two systems: Sonix RP + L14-5/60 (center ~6.3 MHz; RF 40 MHz) and GE LOGIQ E9 + ML6-15 (center ~7 MHz; RF 50 MHz); phantom normalization per-system; ACE via reference phantom method; −6 dB bandwidth fit for MBF/SI/SS	Leave-one-out CV at subject level; random undersampling to balance classes for FLD/SVM (K-NN also reported on unbalanced data); sequential forward feature selection	Not reported	Early-monitoring generalization with mixed hardware; standardized normalization across devices
Dasgupta et al., 2020 [23]	Single large cohort; no external site	Pre-treatment binary R/NR from clinical/pathological endpoint	Sonix RP; linear probe; center ~6.5–7 MHz; RF 40 MHz; QUS parametric maps (MBF/SS/SI + BSC model) → textures → texture-derivatives; phantom normalization; attenuation compensation applied	Cross-validated training/evaluation with repeated sub-sampling for stability; SVM/K-NN/FLD evaluated (SVM best in paper); leakage avoided by subject-level splits	Reported that model-predicted groups mirrored actual RFS in follow-up (prognostic separation)	A priori baseline model for decision support prior to NAC start
Dobruch-Sobczak et al., 2019 [24]	Single site pilot; no external set	Pathology after NAC: cellularity reduction and residual size; R/NR derived from histology	RF acquisition with clinical US; integrated backscatter/scattering coefficient computed; serial scans before and ~1 week after each NAC cycle; phantom-referenced estimation	ROC-based discrimination of early cycles; small N; no formal ML CV	Early cycles predicted final outcome (AUC ~0.82 by dose-2/3 reported)	Early European feasibility using scattering coefficient framework
Sannachi et al., 2023 [25]	Large single site; internal train/val/test split	Baseline (pre-Tx) binary R/NR; subtype included in labeling model	RF-based QUS maps at baseline; phantom-normalized spectra; attenuation-corrected; radiomics + texture-derivatives + subtype features	Supervised ML ensemble; subject-level train/validation/test split (reported in paper); calibration assessed; CI reported	Not reported	A priori hybrid QUS+subtype approach for baseline decisioning
Falou et al., 2024 [26]	Single site with unseen test subset	Baseline R/NR at surgery; subgroup OS/RFS curves shown	RF-based multi-parametric QUS maps; phantom normalization; attenuation correction; transfer-learning on QUS maps	Train/validation with separate unseen test set; TL-CNN features + classical classifier; class performance by precision/recall reported	Survival curves (OS/RFS) provided by clinical groups; QUS prediction reported alongside	Implementation of transfer learning on baseline QUS maps
Dasgupta et al., 2024 [27]	Single-institution randomized feasibility trial	Final response at surgery; week-4 QUS model used to predict early response for adaptation	Sonix RP (L14-5/60, ~6.5 MHz) or GE LOGIQ E9 (ML6-15, ~6.9 MHz); standard RF capture; serial baseline/W1/W4; phantom normalization; attenuation-aware spectral processing	Phase-2 RCT (1:1), stratified by hormone-receptor status; observational vs. experimental (adaptive) arms; model pre-trained on prior 100-pt cohort; prospective validation (week-4 accuracy ~98% stated in manuscript)	Not a survival study; primary = feasibility and prospective predictive performance	QUS-guided adaptive NAC allowed oncologist-directed changes (e.g., early taxane, intensification, or early surgery); 3/5 predicted NR adapted → final responders
Taleghamar et al., 2022 [28]	Single site with internal test set	Baseline R/NR at surgery	RF-based multi-parametric QUS maps at pre-Tx; phantom normalization; attenuation-corrected spectra as DL inputs	CNN with defined train/validation/test split; reports test accuracy/AUC for held-out set	Not reported	Demonstrated deep-learning feasibility at pre-Tx for a priori prediction

RF, radiofrequency; MBF/SS/SI, mid-band fit/spectral slope/spectral intercept; BSC, backscatter coefficient; ASD/SAS, average scatterer diameter/size; AAC/ACE, acoustic attenuation/acoustic concentration or attenuation-coefficient estimate (per study usage); CV, cross-validation; LOPO/LOOCV, leave-one-patient/leave-one-out cross-validation; R/NR, responder/non-responder; SVM/K-NN/FLD, support vector machine/k-nearest neighbors/Fisher’s linear discriminant; TL, transfer learning; CNN, convolutional neural network; KM, Kaplan–Meier; OS/RFS, overall/recurrence-free survival; AUC, area under the ROC curve; NR, not reported.

**Table 4:** Risk-of-bias (ROB) summary and key limitations.

Study (Year) [Ref.]	Patient Selection	Index Test (Feature Leakage Prevention, Blinding)	Reference Standard (Response Definition)	Flow and Timing (Intervals, Attrition)	Analysis (CV/Validation, Class Imbalance, Calibration)	Overall ROB	Key Limitation(s)/Notes
Tran 2016 [17]	SC	SC	SC	L	H	H	Single-site convenience cohort; no external validation; multivariable modeling without clear nested CV; per-timepoint modeling may inflate optimism.
Tadayyon 2016 [18]	SC	SC	SC	L	SC	SC	LOPO CV at patient level; sequential forward feature selection (nesting not explicitly stated); composite responder definition.
Sadeghi-Naini 2017 [19]	SC	SC	SC	L	SC	SC	Heterogeneity-focused monitoring; internal CV only; limited reporting of blinding and calibration.
Sannachi 2018 [20]	SC	SC	SC	L	SC	SC	Multiclass SVM with subject-level CV; no external validation; responder categories derived from clinical+pathologic assessment.
DiCenzo 2020 [21]	SC	SC	SC	L	SC	SC	Multi-institution pooling but no held-out external site; K-NN best on internal CV; AUC NR at baseline; calibration NR.
Quiaoit 2020 [22]	SC	SC	SC	L	SC	SC	Mixed hardware; subject-level LOOCV; random undersampling used; feature selection details limited; external validation NR.
Dasgupta 2020 [23]	SC	L/SC	SC	L	SC	SC	Subject-level splits stated to avoid leakage; repeated subsampling; prognostic separation noted but external validation NR; calibration limited.
Dobruch-Sobczak 2019 [24]	H	SC	SC	L	H	H	Small pilot; ROC analyses without formal CV; very limited sample size; high analysis ROB.
Sannachi 2023 [25]	SC	L/SC	SC	L	SC	SC	Internal train/validation/test split; calibration and CIs reported; still single-center without external site.
Falou 2024 [26]	SC	L/SC	SC	L	SC	SC	Transfer learning with separate unseen test set; external site NR; per-class metrics reported; calibration NR.
Dasgupta 2024 (RCT) [27]	L	L/SC	L	L	SC	SC	Prospective randomized feasibility with pretrained model; strong design for flow/timing; external multi-site validation NR; primary endpoint = decision impact.
Taleghamar 2022 [28]	SC	SC	SC	L	SC	SC	DL with internal train/val/test splits; external validation and calibration NR; reporting of class balance handling limited.

Abbreviations: L = low risk; SC = some concerns; H = high risk; NR = not reported; LOPO = leave-one-patient-out; CV = cross-validation; DL = deep learning; SVM = support vector machine; R/NR = responder/non-responder; AUC = area under the ROC curve.

## Data Availability

Not applicable.

## Supplementary Materials

The following supporting information can be downloaded at: https://www.mdpi.com/article/10.3390/diagnostics16030425/s1, Supplementary Table S1: PRISMA Checklist. Reference [41] is cited in the supplementary materials.
